# Drying the leaves of *Perilla frutescens* increases their content of anticancer nutraceuticals

**DOI:** 10.1002/fsn3.993

**Published:** 2019-03-18

**Authors:** Natsuko Kagawa, Hiroya Iguchi, Masahumi Henzan, Mitsumasa Hanaoka

**Affiliations:** ^1^ Center for Environment, Health and Field Sciences Chiba University Chiba Japan; ^2^ Division of Applied Biological Chemistry, Graduate School of Horticulture Chiba University Chiba Japan

**Keywords:** anticancer, drying, nutraceutical, *Perilla frutescens*

## Abstract

A regular intake of plant‐derived bioactive agents has gained popularity because of the health benefits. Fresh leafy greens, however, normally have a low concentration of such bioactive agents. In this study, we found that drying markedly affected the accumulation of secondary metabolites and that dried leaves of *Perilla frutescens* L. (perilla) contained more anticancer flavonoids than fresh leaves. Drying is a major method of food preparation, particularly for plant‐based foods, but the quality of the bioactive agents contained in the fresh and dried leaves of perilla has received only scant attention. Quantitative analysis of the concentrations of perillaldehyde, rosmarinic acid, apigenin, luteolin, 4‐hydroxyphenyllactic acid, and 4‐coumaric acid, some of which are known as nutraceuticals, revealed that the effect of drying significantly increased apigenin (28‐fold) and luteolin (86‐fold), but decreased rosmarinic acid in all leaf stages. We examined the positive effect on flavonoid levels on perilla leaves and confirmed that, by comparison with fresh perilla leaves, the dried leaves contained greater concentrations of anticancer flavonoids regardless of variety, form, or manner of cultivation. This indicates that drying can significantly increase the level of flavonoids in perilla leaves without a loss of flavor. Therefore, drying is a simple and effective method to improve the concentrations of bioactive agents, which increases the intake of beneficial substances derived from herbs and edible plants. This finding serves as a method for the supply of raw plant materials rich in bioactive agents that are suitable for labeling as edible nutraceuticals.

## 1 INTRODUCTION

Fresh leaves of *Perilla frutescens* L. Britton (perilla) are used as a culinary herb in East Asian countries such as Japan, Korea, and China (Yu, 1997). Perilla is an annual short‐day plant that belongs to the Lamiaceae family along with many aromatic edible plants such as mint, basil, rosemary, sage, oregano, and thyme. In Asian countries, the dried leaves of perilla are used as raw material for crude drugs because they contain potent bioactive substances.

In 1979, Stephen DeFelice defined nutraceuticals as substances that could be a food or a part of a food that provides medical and health benefits such as the prevention and treatment of disease (Brower, 1998; Gupta, Kim, Prasad, & Aggarwal, 2010). Zeisel (1999) proposed that nutraceuticals be defined as diet supplements that deliver a concentrated form of bioactive agents from a food, presented in a nonfood matrix, and used to enhance health in dosages that exceed those that could be obtained in normal foods. In his proposal, the culinary herbs, such as those used in designer foods, should not be recognized as nutraceuticals, diet supplements, or functional foods. Many bioactive agents, however, have been identified in the herbs and edible plants that are considered designer foods. We suppose that plant materials rich in bioactive agents should be recognized as nutraceuticals.

Perilla leaves contain many important bioactive agents and secondary metabolites such as perillaldehyde (PA), rosmarinic acid (RA), apigenin (AG), and luteolin (LT). PA is an original monoterpene and a major compound of the essential oil of perilla (Ito, Toyoda, & Honda, 1999). PA has a unique and faintly sweet flavor and imparts antimicrobial and antidepressant activities (Igarashi & Miyazaki, 2013; Ito, Nagai, Oikawa, Yamada, & Hanawa, 2011). The content of PA in perilla leaves for clinical use is defined in the Japanese pharmacopoeia (Ministry of Health, Labour, & Welfare of Japan, 2017). RA is a phenylpropanoid of polyphenols that is abundant in Lamiaceae's plants such as savory, thyme, oregano, mint, and lemon balm (Petersen, 2013; Vladimir‐Knežević et al., 2014). RA possesses anti‐allergic, anti‐inflammatory, and antioxidant activities and has recently been identified as a potential treatment for Alzheimer's disease (Hamaguchi, Ono, Murase, & Yamada, 2009; Ono et al., 2012; Osakabe et al., 2004; Takano et al., 2004). The content of RA in perilla stems and fruits for clinical use is defined in the Chinese Pharmacopoeia (2015). AG and LT are major plant flavonoids and are widely found in many medicinal plants, vegetables, and fruits. AG and LT possess antioxidant, anti‐inflammatory, and anticarcinogenic activities. AG has been reported for DNA protective activity against UV‐B‐induced DNA damage in skin cells and mice (Das, Das, Paul, Samadder, & Khuda‐Bukhsh, 2013; George, Dellaire, & Rupasinghe, 2017). LT has been reported for its apoptosis potential in many types of cancer cells and in multidrug‐resistant cancer cells by inducing cell‐cycle arrest and apoptosis via intrinsic signaling pathways (George et al., 2017; Rao, Satelli, Moridani, Jenkins, & Rao, 2012). Flavonoids including AG and LT are popular nutraceuticals due to their potential for the prevention and treatment of cancers (George et al., 2017; Gupta et al., 2010; Liu‐Smith & Meyskens, 2016; Patel, Shukla, & Gupta, 2007).

Drying is a major method of food preparation, particularly for plant‐based foods, but it also has a large impact on the secondary metabolism of harvested plants. When applied during the flowering stages of basil leaves (Mandoulakani, Eyvazpour, & Ghadimzadeh, 2017) and during the developmental stage in grape berries, drought stress results in increased levels of gene expression and compounds involved in the phenylpropanoid pathways (Savoi et al., 2016). Our hypothesis is that the secondary metabolisms of these plants, as well as their secondary metabolites, respond to the drying process in varied ways. Drying methods have been evaluated in various ways to minimize the loss of nutrition from fresh plant materials during processing. We realized, however, that few studies have investigated the effect that drying the leaves of perilla can exert on the content of potent bioactive agents that include PA, RA, AG, and LT, although these are known to be responsible for the plant's medicinal efficacy and for its ability to enhance health and fitness. As previously mentioned, the medicinal substances in perilla have the potential to prevent and treat disease, which has recently made them popular nutraceuticals in Japan. These facts prompted the questions of whether fresh or dried leaves would be more suitable as raw material for nutraceuticals in order to avoid the loss of secondary metabolites, as well as what would be the advantages of dried leaves other than long‐term preservation, and, finally, how drying affects the content of particular substances in perilla leaves.

This article describes the effects that the drying of perilla leaves exert on the bioactive agents PA, RA, AG, and LT with a focus on their metabolic changes during each leaf stage. PA and other compounds in perilla essential oil are volatile and have a low boiling point, which means they are easily evaporated under conditions of reduced pressure or increased temperature. Therefore, drying was conducted in a laboratory under atmospheric pressure and air conditioning (<25°C) without light. Quantitative analyses of bioactive agents were conducted via high‐performance liquid chromatography (HPLC) using a photodiode array (PDA) and liquid chromatography–mass spectrometry (LC‐MS). In addition, 4‐hydroxyphenyllactic acid (4HL) and 4‐coumaric acid (4CA) were also subjected to quantitative analysis in order to qualify the biosynthetic activity toward RA, AG, and LT. Herein, we describe the extent to which bioactive agents were changed in the perilla herb during a simple drying process and discuss how the drying may influence the secondary metabolism in each plant. This information should be valuable for researchers working in the development of plant‐based foods and drugs.

## 2 MATERIALS AND METHODS

### 2.1 Perilla materials

#### 2.1.1 Cultivation and sampling

Green perilla (*P. frutescens* var. *crispa* f. *viridis*, Takii & Co., Ltd., Kyoto, Japan) seeds were sown on soils generally used for the growth of vegetables (Kanuma Kosan Co. Ltd., Tochigi, Japan) in plastic pots placed in a cultivation room. The light intensity was set at approximately 80 µmol m^−2^ s^−1^ (the unit of µmol m^−2^ s^−1^ represents photosynthetic photon flux density) with a photoperiod of 16 hr per day provided by cool white fluorescent lamps. Water was provided once per week and was supplemented with a 1/200 dilution of HYPONeX (HYPONeX JAPAN Co. Ltd., Osaka, Japan) once every two weeks. Air temperature was set at 23°C/18°C during light/dark periods. After germination, 10 seedlings were cultivated for 12 weeks and grew to plants that each had eight pairs of leaves. Among them, four pairs of true leaves (the 4th–7th) from the middle regions of each plant were selected as samples and subjected to analytical experiments. All leaf samples were harvested at the same time with great care to avoid any wounding of the leaves, and the fresh weight of each leaf was immediately measured. The two leaves of a single pair will be the same age and at the same stage of growth, which means the appearances of plant growth and metabolism will be approximate. Then, one leaf from each pair was dried and the other remained fresh to allow for accurate comparisons.

#### 2.1.2 Leaf stage

With the exception of cotyledons, the seven pairs of true leaves were numbered from the ground to the top as the first‐to‐seventh stages of leaf development (leaf stage). Cotyledons were eliminated from the experiments because they differed in their plant growth stages. Four pairs of true leaves (the 4th–7th) from the middle regions of each plant were selected as samples and subjected to analytical experiments. One leaf of each pair of true leaves was used as a fresh leaf sample for analysis and the other leaf was used for the preparation of a dried leaf sample.

#### 2.1.3 Fresh leaf samples

A fresh leaf was placed into a polypropylene tube with a metalcorn, and the tube was then frozen in liquid nitrogen and ground using a Multibeads Shocker (Yasui Kikai Co. Ltd., Osaka, Japan). The metalcorns were removed, and the crushed leaf samples were stored at −80°C until they were subjected to analysis along with the dried leaf samples.

#### 2.1.4 Dried leaf samples

Dried leaf samples consisted of the whole leaf. Drying was accomplished by spreading the leaves in darkness under atmospheric pressure at ambient temperature that ranged from 18 to 23°C for a period of three weeks. Subsequently, the weight of the dried leaves was measured. Dried leaf samples were pulverized using the µT‐01 Beads Crusher (Taitec, Saitama, Japan), and the steel beads were removed before the next extraction step.

### 2.2 Quantitative analysis of PA, RA, AG, and LT

#### 2.2.1 Extraction

Extraction was conducted according to the Japanese Pharmacopoeia (Ministry of Health, Labour, & Welfare of Japan, 2017) and a method described in a previous article (Lu et al., 2017) with modifications. A sample, ca 20 mg of a dry leaf and ca 150 mg of a fresh leaf, was weighed accurately and transferred to a 1.5‐ml tube. Methanol (1 ml) was added, mixed for 10 min at 2,000 rpm and 15°C using an Eppendorf ThermoMixer (Hamburg, Germany), and centrifuged for 5 min. To the residue, methanol (1 ml × 2) was added, and the same extract manner was performed twice. The extracts (about 3 ml) were combined and transferred to a 5‐ml volumetric flask and diluted with methanol to a 5 ml total volume. The solution was filtered through a 0.22‐µm nylon syringe filter (Shimadzu GLC Ltd., Tokyo, Japan) to prepare the samples for HPLC or LC‐MS.

#### 2.2.2 PA and RA concentration

High‐performance liquid chromatography analysis of PA and RA was conducted according to methods described in previous literature with modifications (Jirovský et al., 2007; Lu et al., 2017; Natsume, Muto, Fukuda, Tokunaga, & Osakabe, 2006; Öztürk, Duru, İnce, Harmandar, & Topҫti, 2010; Sevindik et al., 2015; Vladimir‐Knežević et al., 2014). HPLC was performed on a Shimadzu LC‐20A Prominence system equipped with a SIL‐20AC autosampler and a SPD‐20A PDA detector using LabSolutions software (Shimadzu, Kyoto, Japan). The HPLC conditions for PA were TSKgel ODS‐80T_M_ column (5 µm, 4.6 × 150 mm) (Tosoh, Tokyo, Japan); temperature, 40°C; flow rate, 1.4 ml/min; run time, 18 min; detector wavelength, 230 nm; mobile phase, 40% acetonitrile; and injection volume, 10 µl. The HPLC conditions for RA were TSKgel ODS‐80T_M_ column (5 µm, 4.6 × 150 mm) (TOSOH, Tokyo, Japan); temperature, 40°C; flow rate, 1.4 ml/min; run time, 35 min; detector wavelength, 330 nm; mobile phase, 15% acetonitrile/0.1% formic acid; and injection volume, 10 µl. The PA and RA standards were dissolved in methanol and used to identify the chromatographic retention times and UV spectra. The quantification of integrated peak areas of the samples allowed comparison with the calibration curves of the corresponding standard solutions. The PA or RA content per fresh leaf weight (hereafter, PA or RA concentration) was estimated by dividing the PA or RA content in the samples by the weight of the fresh sample. Therefore, for dried samples, the PA or RA concentrations were estimated by dividing the PA or RA content in dried samples by the weight of the fresh sample before drying.

#### 2.2.3 AG, LT, 4HL, and 4CA concentrations

Liquid chromatography–mass spectrometry analysis of AG and LT was conducted according to a method described in previous literature (Lu, Takagaki, Yamori, & Kagawa, 2018) with modifications. A LCMS‐2020 mass spectrometer (MS) equipped with an electrospray ionization (ESI) source operating in negative mode was used for identification and quantification of AG, LT, 4HL, and 4CA by chromatographic data processed using LabSolutions software (Shimadzu, Kyoto, Japan). The HPLC conditions for AG and LT were XBridge BEH C18 column (3.5 µm, 2.1 × 150 mm; Waters, MA, USA); temperature, 40°C; flow rate, 0.2 ml/min; run time, 15 min; mobile phase, 30% acetonitrile/0.1% formic acid; and injection volume, 1 µl. The HPLC conditions for 4HL and 4CA were XSelect HSS T3 column (3.5 µm, 2.1 × 75 mm; Waters); temperature, 30°C; flow rate, 0.2 ml/min; run time, 20 min; mobile phase, 10% acetonitrile/0.1% formic acid; and injection volume, 1 µl. The eluent was passed to the electrospray source. A capillary voltage of 3.5 kV was used in the negative ion mode. Nitrogen was used as drying gas with a flow rate of 15 L/min and as nebulizing gas with a flow rate of 1.5 L/min. The desolvation line temperature was set at 250°C. The ion trap was operated in full scan mode from *m/z* 50 to 1,000 and selected ion monitoring (SIM) mode with *m/z* 269 for a molecular ion [M−H]^−^ of AG, *m/z* 285 for a molecular ion [M−H]^−^ of LT, *m/z* 181 for a molecular ion [M−H]^−^ of 4HL, and *m/z* 163 for a molecular ion [M−H]^−^ of 4CA. The AG and LT standards were dissolved using a scant amount of THF followed by dilution with methanol, and the standards of 4HL and 4CA were dissolved in methanol to prepare the standard solutions before their use in identification and quantification. The results of AG, LT, 4HL, and 4CA concentrations were calculated and expressed in the same manner as those of PA and RA.

#### 2.2.4 Chemicals

The PA, RA, methanol (LC‐MS grade), and water (LC‐MS grade) were purchased from FUJIFILM Wako Pure Chemical Corporation (Osaka, Japan). The AG, LT, 4HL (DL‐4‐hydroxyphenyllactic acid), and 4CA (*trans*‐4‐coumaric acid) were obtained from Tokyo Chemical Industry Co., Ltd. (Tokyo, Japan). Acetonitrile (HPLC grade) was obtained from Sigma‐Aldrich, Japan (Tokyo, Japan). Formic acid was purchased from Kanto Chemical Co., Inc. (Tokyo, Japan).

### 2.3 Statistical analysis

Data were subjected to independent sample testing using SPSS 25.0 software (IBM Japan, Tokyo, Japan). The Levene's test was conducted for equality of variances. Significant differences between the means of fresh and dried leaves were calculated for each leaf stage using Student's *t* test (*p* < 0.05). The test was conducted to determine the significance of drying. All experiments were repeated twice for five replications.

## 3 RESULTS

### 3.1 Total concentrations of PA, RA, 4HL, 4CA, AG, and LT

The data reported in this study are based on a wet basis wherein concentrations (µg g^−1^/mg g^−1^) are expressed as the content per unit of fresh (pre‐dry) leaf weight. The fresh leave samples contained a higher water content at 74 ± 1.7% (the mean ± standard error, *n* = 20). The concentration of each dried leaf sample was estimated by dividing the content in the dried leaf sample by the weight of the pre‐dry leaf sample that was measured before drying.

Table 1 shows the changes in the concentrations of PA, RA, 4HL, 4CA, AG, and LT in every leaf sample to allow comparisons between the fresh and dried leaves. The concentration of PA slightly decreased after drying (−6%), but the reduction was not significant. The concentration of RA largely decreased after drying (−81%, *p* < 0.001). The reduction of 4HL was observed after drying (−66%, *p* < 0.001). The concentration of 4CA, however, showed a marked increase after drying (fourfold, *p* < 0.001). The concentration of AG, however, was significantly increased after drying (16‐fold, *p* < 0.001). The effect on LT was also significant with a 109‐fold increase in concentration in the dried leaves (*p* < 0.001).

**Table 1:** Concentrations of compounds extracted from fresh and dried leaves of perilla

Concentrations^a^	Fresh	Dry
Perillaldehyde	928 ± 165	868 ± 143
Rosmarinic acid	3,140 ± 318	609 ± 170***
4‐Hydroxyphenyllactic acid	8.07 ± 1.00	2.76 ± 0.279***
4‐Coumaric acid	0.233 ± 0.0486	0.939 ± 0.151***
Apigenin	0.419 ± 0.0809	6.68 ± 0.971***
Luteolin	0.154 ± 0.0150	16.8 ± 1.89***

a Concentrations (µg/g) are expressed as the content per unit of fresh (predry) leaf weight. Values are recorded as the mean ± standard error (*n* = 14‒20). Significant differences between fresh and dried leaves are indicated by ***p < 0.001, according to Student's *t* test.

### 3.2 Local concentrations of PA, RA, 4HL, 4CA, AG, and LT based on leaf stage

**Figure 1:**
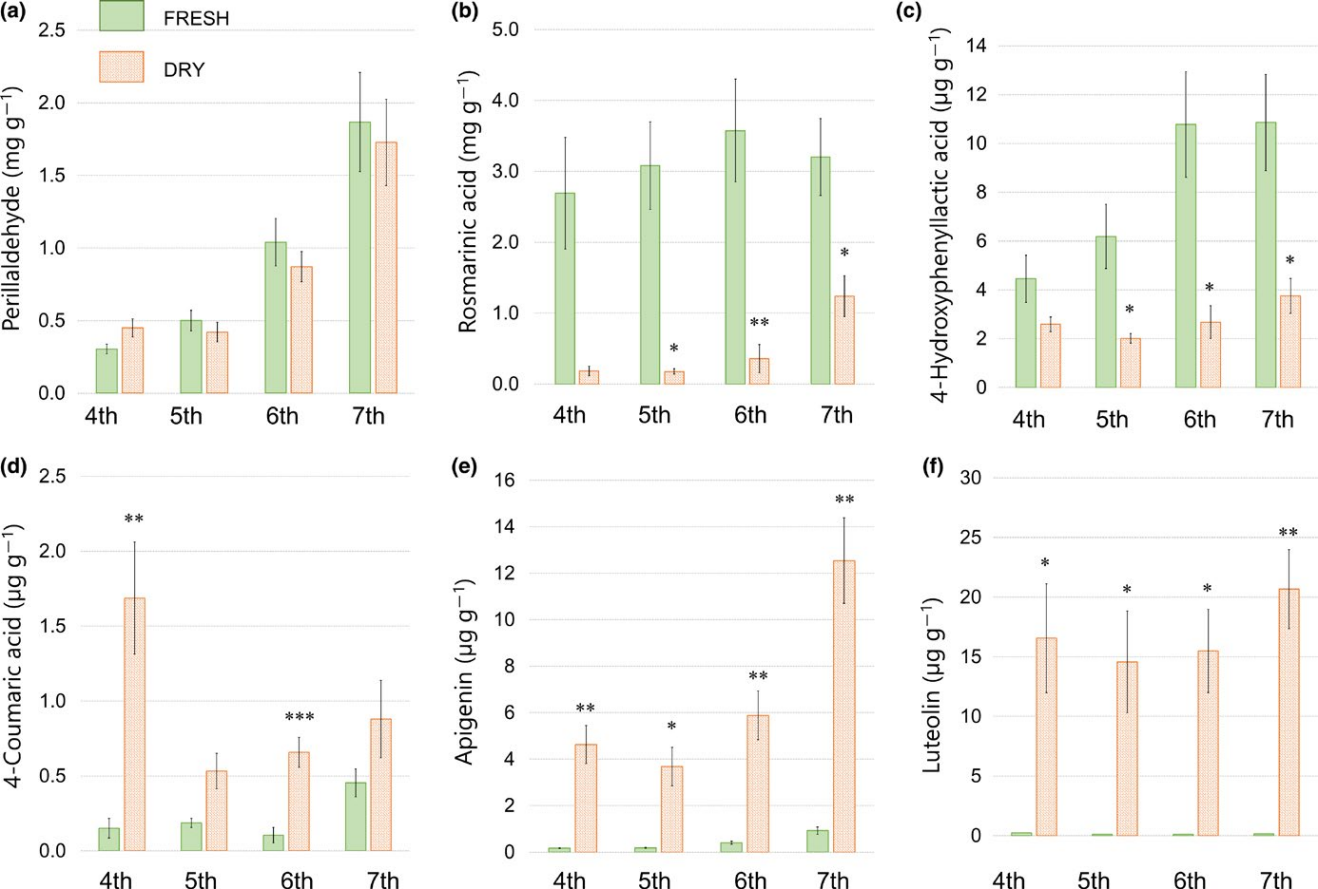
Comparing the concentrations of fresh and dried leaves based on leaf stage. (a) Perillaldehyde; (b) rosmarinic acid; (c) 4‐hydroxyphenyllactic acid; (d) 4‐coumaric acid; (e) apigenin; (f) luteolin. Concentrations are expressed as content per unit of fresh (pre‐dry) leaf weight. Values represent the mean ± standard error (*n* = 4‒5). For each leaf stage, significant differences between fresh and dried leaves are indicated by *p < 0.05, **p < 0.01, ***p < 0.001, according to Student's *t* test

The changes in the concentrations of each compound between fresh and dried leaves on the leaves situated from the fourth to the seventh leaf stages are shown in Figure 1. The concentration of PA increased locally on the fourth‐stage dried leaves (150%), but decreased on the fifth to the seventh‐stage dried leaves (−16%, −16%, −7%, respectively, Figure 1a). Significant depletion occurred in the concentration of RA for every leaf stage: −94% (*p* < 0.05) for the fifth‐stage dried leaves; −90% (*p* < 0.01) for the sixth‐stage dried leaves; and −61% (*p* < 0.05) for the seventh‐stage dried leaves (Figure 1b). The concentration of 4HL showed a trend similar to that of RA: −68% (*p* < 0.05) for the fifth‐stage dried leaves; −75% (*p* < 0.05) for the sixth‐stage dried leaves; and −65% (*p* < 0.05) for the seventh‐stage dried leaves (Figure 1c). The concentration of 4CA was increased in every leaf stage with significant increases for the fourth‐ (11‐fold, *p* < 0.01) and sixth‐stage (sevenfold, *p* < 0.001) dried leaves (Figure 1d). The accumulations were extremely high for AG and LT in the dried leaves for all leaf stages (Figure 1e,f). The fourth‐stage leaves showed increases in AG and LT (27‐fold, *p* < 0.01; 72‐fold, *p* < 0.05, respectively). The fifth‐stage leaves showed a 20‐fold increase for AG (*p* < 0.05) and a 120‐fold increase for LT (*p* < 0.05). The sixth‐stage leaves recorded a rise in the levels of AG (15‐fold increase, *p* < 0.01) and LT (120‐fold increase, *p* < 0.05). The seventh‐stage dried leaves recorded the highest levels of AG and LT with increases of 14‐ (*p* < 0.01) and 150‐fold (*p* < 0.01), respectively.

### 3.3 States of variety, form, cultivation, and condition

In order to generalize these results concerning AG and LT accumulation, we shopped markets to collect commercially available perilla leaves of several varieties from different methods of cultivation to conduct further surveys. These additional results appear in Table 2. The perilla materials listed in Table 2 were composed of five varieties, one green form and four red forms, and were cultivated in four manners that differed from those described in Section 2.1.1 (Supporting information Table S1). These had been grown in indoor cultivation systems or outdoor at various farms. The fresh leaves were obtained 3–5 days after harvest and were then analyzed. The fresh leaves were dried by repeating the methods described in 2.1.4. The concentration of AG increased significantly following drying (54‐fold, *p* < 0.001), and the concentration of LT had increased in the dried leaves (37‐fold, *p* < 0.001; Table 2).

**Table 2:** Flavonoid concentrations in fresh and dried leaves collected from markets

Concentrations^a^	Fresh	Dry
Apigenin	0.0708 ± 0.037	3.85 ± 1.9***
Luteolin	0.134 ± 0.081	4.97 ± 2.1***

a Concentrations (µg/g) are expressed as content per unit of fresh (predry) leaf weight. Values represent the mean ± standard error (*n* = 17). Significant differences between fresh and dry leaves are indicated by ***p < 0.001, according to Student's *t* test.

## 4 DISCUSSION

**Figure 2:**
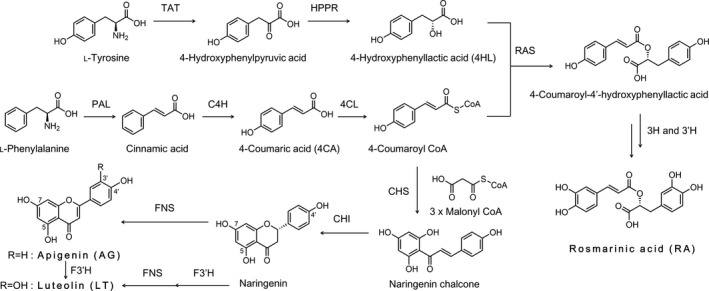
Proposed biosynthetic pathways for rosmarinic acid, 4‐hydroxyphenyllactic acid, 4‐coumaric acid, apigenin, and luteolin in the perilla plant. Shown here are the core enzymes involved in the biosynthesis of phenylpropanoid and flavone skeletons. CHI: chalcone isomerase; CHS: chalcone synthase; 4CL: 4‐coumaric acid: CoA ligase; C4H: cinnamic acid 4‐hydroxylase; 3H and 3′H, hydroxycinnamoyl‐hydroxyphenyllactate 3‐ and 3′‐hydroxylases; F3′H: flavonoid 3′‐hydroxylase; FNS: flavone synthase; HPPR: hydroxyphenylpyruvate reductase; hydroxycinnamoyl‐CoA: hydroxyphenyllactate hydroxycinnamoyl transferase; PAL: phenylalanine ammonia‐lyase; RAS: rosmarinic acid synthase; TAT: tyrosine aminotransferase

Studies on a molecular basis have promoted an understanding of the biosynthetic pathways of RA, AG, and LT. The enzymes involved in the biosynthesis of RA from amino acids were unraveled in suspension cultures of *Anchusa officinalis* (Boraginaceae) and *Coleus blumei* (Lamiaceae) by Petersen and Simmonds, (2003) and Petersen (2013). Flavonoid biosynthesis, which includes the core pathways, has been studied in different plants (Bashandy et al., 2015; Dao, Linthorst, & Verpoorte, 2011). Moreover, the entire transcriptome map of perilla leaves has been reported and the expression of genes involved in the biosynthetic pathways of flavonoids and phenylpropanoids has been clarified (Fukushima, Nakamura, Suzuki, Saito, & Yamazaki, 2015). Thus, the proposed biosynthetic pathways in perilla are shown in Figure 2. The core structure of RA is an ester with molecules of 4HL and 4‐coumaroyl CoA. 4HL is derived from L‐tyrosine, and 4‐coumaroyl CoA is synthesized from 4CA in the phenylpropanoid pathway. The pathway from L‐phenylalanine to 4‐coumaroyl CoA is used for the biosynthesis of flavonoids. AG and LT are synthesized via the polyketide pathway, which starts with the condensation of 4‐coumaroyl CoA and three molecules of malonyl‐CoA by the enzyme chalcone synthase (CHS, EC 2.3.1.74) to yield naringenin chalcone (Dao et al., 2011). The chalcone is isomerized to a (2*S*)‐flavanone naringenin by chalcone isomerase (CHI), and the naringenins are transformed to flavones AG and LT by flavone synthase (FNS) and flavonoid 3′‐hydroxylase (F3′H).

As shown in Table 1 and Figure 1, drying resulted in positive effects in the concentrations of 4CA, AG, and LT and in negative effects in the concentrations of 4HL and RA. In other words, the production levels of 4CA, AG, and LT molecules were increased and 4HL and RA accumulations were simultaneously suppressed during drying. It seems that the flavone biosynthetic pathway, which includes 4CA biosynthesis, was activated by the stress of drying and that the RA biosynthetic pathway, which includes 4HL biosynthesis, was deactivated. Decreased accumulation in RA molecules may have been caused by increases in the consumption of RA and its biosynthetic precursors. Given that 4‐coumaroyl CoA, as a necessary key precursor for RA and flavones, had appeared in both of the pathways branching to RA and flavones, increased production of flavones required a greater flow of 4‐coumaroyl CoA into the polyketide pathway to react with malonyl‐CoA, which could have induced depletion of the 4‐coumaroyl CoA involved in the esterification with 4HL by rosmarinic acid synthase (RAS; E.C. 2.3.1.140). Competition for precursors and enzymes is common in plant metabolism, and the phenomenon can create complexities. We revealed, however, that stimulation by the drying process changes the secondary metabolites in a plant's biosynthetic pathways. RA biosynthesis was a major consumer of 4CA in fresh leaves. During the drying process, however, RA production was diminished and 4HL was depleted, while the biosynthesis of flavonoids required more 4CA for AG and LT production than that required in fresh leaves. This switch could contribute in part to an increase in the accumulation of flavones in correlation with a decreasing supply of RA in drying leaves.

It should be noted that AG and LT characteristically have low solubilities in water, which could result in a disadvantage for fresh materials that would result in lower efficiency during extraction, because fresh materials contain greater amounts of water compared with dried materials. Changes in the appearance of flavones, AG and LT, however, seemed to indicate that drying had a positive influence on plant metabolism and on the production of AG and LT, which ran completely contrary to the influence on RA. The compound RA itself is chemically stable as the ester and the carboxylic acid portion in the structure is not reactive with one another, which enables polymerization—as opposed to the presence of aldehyde in the PA structure. An example was found in the literature whereby the increase in temperature (50°C < 80°C) during the drying of perilla leaves had caused a reduction in the concentration of RA (Okuda, Hatano, Isao, & Nishibe, 1986). In the present study, however, the concentration of PA was not significantly reduced after drying, as shown in Table 1 and in Figure 1, so that this level of heat is apparently suitable for chemicals such as PA that are sensitive to air, light, and temperature. Therefore, the significant decrease in RA accumulation following the drying process was due to plant metabolism rather than to decomposition by chemical reaction. The reduction in RA accumulation in cells meant that RA consumption had overcome RA formation during drying. Otherwise, the RA level in the dried leaves would have remained at the same level of RA in the fresh leaves. We theorized that perilla had used RA as a protectant against the stresses and damages of the drying process (Döring & Petersen, 2014).

The sums of flavones AG and LT were increased significantly (41‐fold) in the dried leaves based on the values calculated from Table 1 and the flavone majority had switched from AG to LT. The ratio of the two compounds (AG/LT) in fresh leaves was 3.2 ± 0.58, but the ratio was switched to 0.42 ± 0.039 in dried leaves with a significant decrease in the amounts (*p* < 0.001). LT is formed from either an equivalent of AG or its corresponding precursor flavanone, naringenin, by hydroxylation at the 3′ position of the aromatic ring by the enzyme F3′H (Figure 2). This switch in flavone accumulation implied that the molecular conversion into LT by F3′H was enhanced as the activity of F3′H was improved during drying.

To support the observation related to the activation of the biosynthetic pathway, we attempted to determine the expression levels of the enzymes in the pathway by measuring the RNA transcript levels. However, we observed a decomposition of the RNAs in the dried leaf cells and could not compare the expression levels between fresh and dried leaves. Therefore, the enzyme activity could not be accurately determined through the expression of proteins extracted from dried leaf cells because of damage sustained during drying.

We compared the data for AG and LT accumulation shown in Tables 1 and 2. Similar effects of drying were observed, as shown by the data summarized in the Tables, in which AG and LT concentrations were highly promoted by drying. There was no change in the ratio (AG/LT) between fresh and dried leaves based on the values calculated from Table 2 (0.62 ± 0.084 and 0.80 ± 0.059, respectively). Drying had a positive effect on the accumulation of AG and LT flavones in perilla plants, and the content level of flavonoids was significantly higher in dried leaves for all states of variety, form, leaf stage, cultivation, and condition.

## 5 CONCLUSIONS

A comparison of the concentrations of PA, RA, AG, and LT in fresh and dried leaves revealed that after drying, AG and LT were increased 28‐ and 86‐fold, respectively, with a 94% decrease in RA. PA was not changed significantly. This proved that drying has a significant impact on phenylpropanoid and flavonoid biosynthesis and accumulation in perilla leaves. We also confirmed that the drying could promote AG and LT accumulation in perilla leaves regardless of variety, form, leaf stage, or manner of cultivation. This finding underscores the use of drying as a method for food processing that can provide raw materials rich in anticancer flavones that are suitable for use as nutraceuticals that contain *P. frutescens*.

## CONFLICT OF INTEREST

The authors have no conflict of interest to declare.

## DATA SHARING AND DATA ACCESSIBILITY

The data that support the findings of this study are openly available in the online version of this article at https://doi.org/10.1002/fsn3.993

## ETHICAL REVIEW

This study does not involve any human or animal testing.

## INFORMED CONSENT

Written informed consent was obtained from all study participants.
