# Supplementary material for: Drying the leaves of Perilla frutescens increases their content of anticancer nutraceuticals
Source: Food Sci Nutr. 2019 Mar 18;7(4):1494–501. doi: 10.1002/fsn3.993 (PMC6475738; doi:10.1002/fsn3.993)
Supplement: Supplementary file 1 [file FSN3-7-1494-s001.docx]

**Table S1.** Eight different perilla materials collected from markets.

| *No.* | *variety* | *form* | *location of farm*  *(province, country)* | *cultivation* |
| --- | --- | --- | --- | --- |
| 1 | a | green | Aichi, Japan | green house |
| 2 | b | red | Chiba, Japan | outdoor |
| 3 | c | red | Saitama, Japan | indoor with LED lighting |
| 4 | d | red | Saitama, Japan | indoor with LED lighting |
| 5 | e | red | Saitama, Japan | indoor with LED lighting |
| 6 | c | red | Hokkaido, Japan | outdoor |
| 7 | d | red | Hokkaido, Japan | outdoor |
| 8 | e | red | Hokkaido, Japan | outdoor |
